# Trauma-induced uterine prolapse in the third trimester: a rare case report

**DOI:** 10.1186/s12884-025-08582-0

**Published:** 2025-12-26

**Authors:** Cenk Soysal, Hatice Merve Başarı

**Affiliations:** 1 Department of Obstetrics and Gynecology, Faculty of Medicine, Kütahya Health Sciences University, Evliya Çelebi Campus on Tavşanlı Road 10. km, Kütahya, 43020 Turkey; 2 Department of Obstetrics and Gynecology, Simav Doç. Dr. İsmail Karakuyu State Hospital, Kütahya, Turkey

**Keywords:** Uterine prolapse, Pregnancy, Trauma, Third trimester, Emergency cesarean section, Obstetric complication, Case report

## Abstract

**Background:**

Uterine prolapse during pregnancy is an extremely rare and challenging obstetric complication, particularly in the third trimester. Trauma-induced prolapse is even less common and can have significant maternal and fetal consequences. Reporting such rare presentations is crucial for guiding clinical management.

**Case presentation:**

We present the case of a 25-year-old gravida 3, para 1 woman at 36 weeks of gestation who was admitted to the emergency department after trauma to the vaginal region. She reported spontaneous rupture of membranes at home prior to admission. On examination, a grade 4 uterine prolapse with a hyperemic and edematous cervix was observed protruding through the vaginal introitus. Laboratory and imaging findings were within normal limits, and there were no signs of retroplacental bleeding or fetal distress. Due to the advanced stage of prolapse and ongoing regular contractions, an emergency cesarean section was performed. The maternal and neonatal outcomes were favorable, and the prolapse resolved completely postpartum.

**Conclusions:**

This case highlights the importance of recognizing and promptly managing trauma-induced uterine prolapse in late pregnancy. It underlines the need for multidisciplinary care and individualized decision-making to ensure optimal maternal and fetal outcomes in such rare clinical scenarios.

## Background

Uterine prolapse during pregnancy is a rare condition, with an estimated incidence of 1 in 10,000 to 1 in 15,000 pregnancies [1]. Although most commonly observed in multiparous women, its etiology remains multifactorial, involving congenital or acquired pelvic floor weakness, repeated vaginal deliveries, short interpregnancy intervals, and connective tissue disorders [2, 3]. The clinical course of uterine prolapse in pregnancy varies widely; complications can include preterm labor, urinary tract infections, cervical ulceration, obstructed labor, and rarely, maternal or fetal morbidity [4, 5].

Most published cases involve spontaneous or chronic uterine prolapse, usually in women with a history of multiple vaginal deliveries or previous pelvic floor injury. For instance, Ba’Abbad et al. [6] reported a case of third-degree uterine prolapse in a term multiparous woman, ultimately managed by cesarean section due to cervical dystocia. Helvacıoğlu and Kahraman Ersoy [7] described a second-trimester case managed successfully with a vaginal pessary, resulting in an uncomplicated vaginal delivery at term. Zeng et al. [8] presented two cases: one with recurrent prolapse in successive pregnancies, and another managed conservatively with a pessary. In all these reports, conservative management and individualized obstetric care were emphasized, and most patients had favorable maternal and fetal outcomes.

Notably, none of the previous cases in the literature were attributed to direct trauma during pregnancy. The present report is, to our knowledge, the first to describe acute uterine prolapse developing in the third trimester following blunt trauma to the vaginal region. This unique case contributes to the existing literature by highlighting trauma as a possible precipitating factor for uterine prolapse during pregnancy and underscores the importance of prompt diagnosis and multidisciplinary management in such rare clinical scenarios.

## Case presentation

**Fig. 1:**
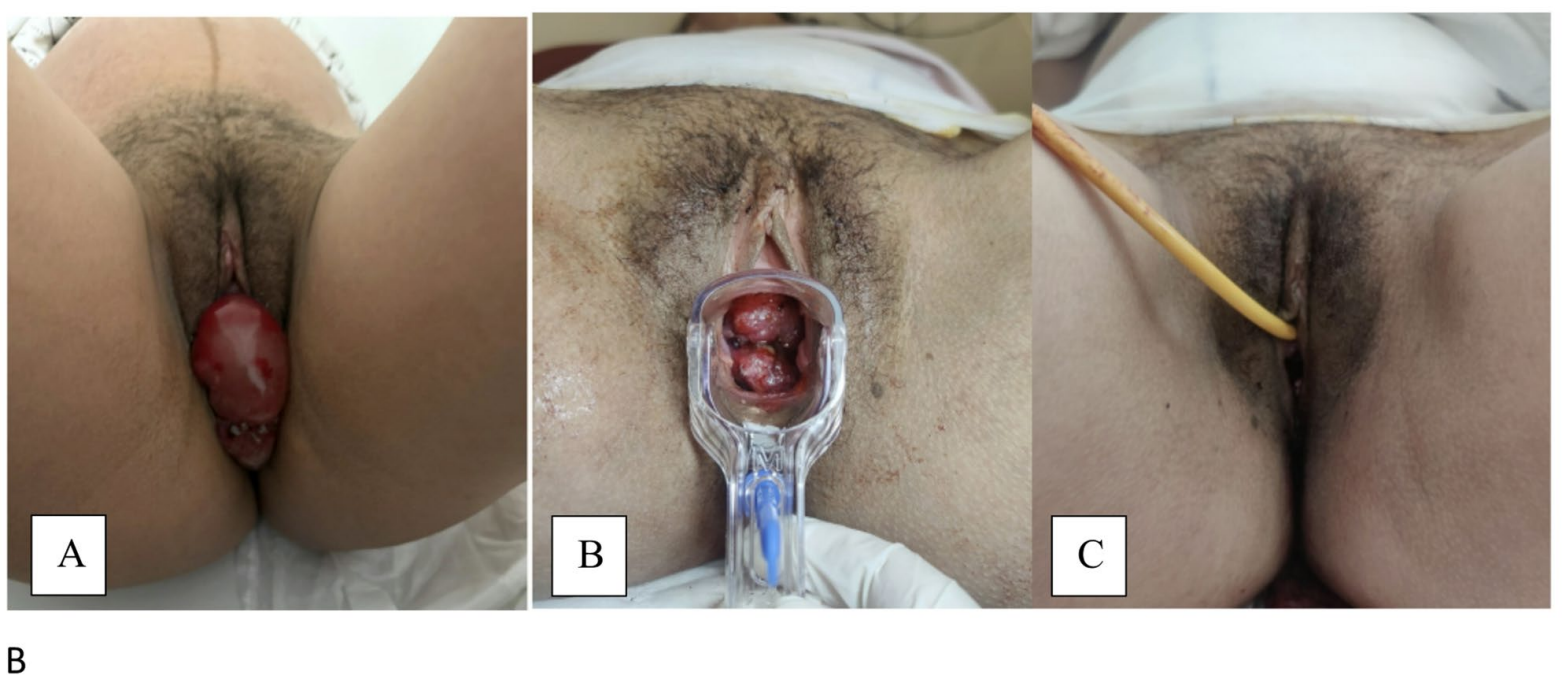
**A**: Preoperative appearance showing advanced uterine prolapse immediately after admission, with a wider-angle photograph including part of the lower abdomen for better visualization of the pregnancy. **B**: Postoperative 6th-hour speculum examination demonstrating regression of the prolapsed tissue. **C**: Postoperative 1st-day perineal evaluation showing complete resolution of the prolapse

A 25-year-old multiparous woman (gravida 3, para 1) at 36 weeks and 1 day of gestation was brought to the emergency department by ambulance following an incident of suspected domestic physical assault. The patient stated that spontaneous rupture of membranes had occurred at home shortly before the event. According to her report, she sustained a direct blunt impact directed between the legs, striking the perineal/vaginal region without involvement of the abdomen. She denied any form of abdominal trauma, and the mechanism of injury was consistent with an isolated perineal blow (Fig. 1A). The patient had no prior history of pelvic trauma, pelvic floor injury, or previous abdominal or gynecologic surgery.

Her obstetric history included no chronic diseases and no prior history of pelvic organ prolapse. She was taking vitamin D, Gynoferrosanol, and Progestan (the latter prescribed during episodes of bleeding). She had not received a two-dose tetanus vaccination; her quadruple test indicated a risk (NTD: 1:156). She was previously referred to perinatology for further evaluation but did not attend the appointment. At 26 weeks of gestation, due to threatened preterm labor, two doses of Celestone were administered. Her oral glucose tolerance test was elevated, for which she was started on 2 units of Levemir daily.

On admission, physical examination revealed no ecchymosis or contusions consistent with blunt trauma to the abdomen or extremities. However, inspection revealed a hyperemic lesion compatible with a bite mark on the lateral neck. Abdominal examination showed regular contractions. Vaginal examination identified a grade 4 uterine prolapse, with the cervix and upper vagina hyperemic, markedly edematous, and protruding through the introitus. The prolapsed tissue was hemorrhagic and positive for a nitrazine (turnusol) test, consistent with ruptured membranes.

Ultrasound assessment revealed a singleton fetus in cephalic presentation, positive fetal heart activity, and a posterior placenta. Biometric measurements were as follows: biparietal diameter 32 weeks, head circumference 31 + 1 weeks, abdominal circumference 33 weeks, femur length 33 + 3 weeks, and estimated fetal weight 2040 g. No retroplacental hematoma or signs of abruption were observed. The amniotic fluid index was 8 cm. Non-stress test demonstrated a reactive fetal heart rate pattern with regular contractions.

Laboratory findings included hemoglobin 13.7 g/dL, hematocrit 40.4%, platelets 161 × 10^9^/L, creatinine 0.33 mg/dL, urea 12.9 mg/dL, ALT 5.5 U/L, AST 15 U/L, sodium 136 mmol/L, potassium 3.9 mmol/L, and normal coagulation parameters (INR 0.97, aPTT 26.7 s, PT 8.82 s). White blood cell count and C-reactive protein levels were within normal limits. Serology (ELISA) was negative. The patient’s blood type was O Rh-positive.

Initial management included placing the patient in a slight Trendelenburg position and attempting gentle manual reduction of the prolapsed cervix. However, due to severe edema, hyperemia, and tissue friability, the cervix could not be successfully reduced or maintained intra-vaginally. Conservative options such as pessary placement were not feasible in the context of acute trauma, ruptured membranes, and active uterine contractions.

Given the presence of advanced uterine prolapse, ongoing regular contractions, and ruptured membranes, an emergency multidisciplinary consultation was performed. Vaginal delivery was not attempted because the cervix was markedly edematous and prolapsed outside the introitus (grade 4), creating a mechanical obstruction and significant risk of cervical trauma, hemorrhage, and failure of progression. Therefore, cesarean delivery was deemed the safest option for both mother and fetus. The patient underwent an emergency cesarean section at 36 weeks and 1 day of gestation. Both maternal and neonatal outcomes were favorable, with the newborn presenting Apgar scores of 8 and 9 at 1 and 5 min, respectively, and the prolapse resolving completely in the immediate postpartum period. At follow-up, the patient’s postpartum course was uneventful, with no recurrence of prolapse or additional complications observed (Figure-1B and 1 C). A final pelvic examination was performed at 48 h postoperatively, confirming complete resolution of the prolapse, after which the patient was discharged.

## Discussion and conclusions

The complete postpartum resolution of the prolapse can be explained by the fact that the etiology was acute, trauma-induced cervical descent rather than a chronic pelvic floor defect. After delivery, the removal of fetal pressure, uterine involution, reduction of cervical edema, and restoration of pelvic support structures allowed the cervix and uterus to return spontaneously to their normal anatomical position.

The literature identifies several risk factors associated with uterine prolapse during pregnancy, including multiparity, congenital pelvic floor laxity, prior obstetric trauma, and short interpregnancy intervals [9]. Pregnancy-related physiological changes—such as increased intra-abdominal pressure, progesterone-mediated ligamentous relaxation, and cervical softening—may further compromise pelvic support in susceptible women [8, 10]. Reported antepartum and intrapartum complications include urinary retention, cervical ulceration, preterm labor, obstructed labor, and, in severe cases, fetal distress or intrauterine infection [8, 11]. Management strategies described in the literature range from conservative measures such as bed rest, Trendelenburg positioning, manual reduction, and pessary placement to operative delivery when the prolapse is severe, irreducible, or complicated by ruptured membranes or active labor [11]. Our case aligns with these observations, demonstrating that trauma-induced acute prolapse can present abruptly and may require individualized and rapid decision-making to prevent maternal–fetal morbidity.

Recent case-based reviews and series emphasize that management of uterine or pelvic organ prolapse during pregnancy should be individualized, with a clear preference for conservative, uterus-preserving strategies whenever feasible. Conservative options include manual reduction of the prolapsed uterus, pessary placement, bed rest, and Trendelenburg positioning, combined with close maternal–fetal surveillance; these approaches have been shown to allow continuation of pregnancy to viability or term in many reports [12, 13]. When prolapse is severe, irreducible, associated with marked cervical edema or trauma, or complicated by ruptured membranes and active labor, several authors recommend operative delivery—most commonly cesarean section—to avoid obstructed labor, cervical laceration, or fetal compromise [14, 15]. In selected women with preexisting advanced prolapse who have completed childbearing, definitive surgical procedures such as cesarean hysterectomy or postpartum hysteropexy may be considered, whereas uterus-sparing approaches are preferred in younger patients who wish to preserve fertility [12, 14]. Our management, favoring emergency cesarean delivery for an acute, irreducible grade-4 prolapse in the setting of trauma and ruptured membranes, is consistent with these contemporary recommendations.

Research indicates that women who experience pelvic trauma can develop a diverse array of pelvic floor dysfunction symptoms, with the most frequently reported issues involving the urinary bladder, bowel, and sexual health [16]. While pelvic organ prolapse (POP) arises from multiple contributing factors, there is occasional evidence suggesting that pelvic trauma may play a role in the progression to advanced POP. Potential pathophysiological mechanisms include disruption of the bony pelvis, damage to the supporting structures of the visceral fascia, neuropathic injuries, and impairment of the pelvic floor musculature [17].

This distinction is clinically significant, as trauma in pregnancy may produce a spectrum of pelvic injuries, but the sudden appearance of a large, hyperemic, and edematous mass at the introitus should prompt consideration of uterine prolapse, not just simple cervical edema. Both conditions can mimic each other, particularly in the context of trauma, making careful clinical assessment and detailed history-taking essential for accurate diagnosis. The risk of misdiagnosis is heightened if clinicians focus solely on the traumatic event or neglect a full gynecological examination.

In our patient, a meticulous examination and focused obstetric history were pivotal in distinguishing between massive cervical edema and true uterine prolapse, thereby enabling the obstetric team to rapidly initiate appropriate, multidisciplinary management. Emergency cesarean delivery was performed due to the severity of the prolapse, ongoing contractions, and ruptured membranes, resulting in a favorable outcome for both mother and neonate. Complete resolution of the prolapse was observed postpartum.

This case expands the clinical spectrum of uterine prolapse in pregnancy, underscoring that acute prolapse can indeed occur as an immediate complication of pelvic trauma. Obstetricians should be aware that in pregnant trauma patients, the sudden appearance of protruding vaginal tissue warrants a high index of suspicion for uterine prolapse, not just cervical swelling. Accurate and early differentiation through careful examination and history can be life-saving, allowing for tailored intervention and prevention of severe maternal-fetal morbidity.

This case underlines several key clinical points. First, any protruding vaginal mass in a pregnant trauma patient should immediately raise suspicion for uterine prolapse, and clinicians must differentiate it from massive cervical edema through meticulous examination. Second, early screening for pregnancy-related pelvic floor instability and careful assessment after trauma may help prevent delayed or missed diagnosis. Third, conservative measures—including manual reduction, Trendelenburg positioning, pessary use, and close maternal–fetal monitoring—should be considered when feasible. Finally, when the prolapse is irreducible, severely edematous, or complicated by ruptured membranes and active labor, cesarean delivery becomes the safest medico-surgical management. Recognizing these principles may reduce maternal–fetal morbidity in similar high-risk scenarios.

## Data Availability

All data generated or analyzed during this case report are included in this published article.

## Abbreviations

BMI: Body mass index
POP: Pelvic organ prolapse
PROM: Premature rupture of membranes
NST: Non-stress test
AFI: Amniotic fluid index
G: Gravida
P: Parity
USG: Ultrasonography
ELISA: Enzyme-linked immunosorbent assay
ALT: Alanine aminotransferase
AST: Aspartate aminotransferase
INR: International normalized ratio
aPTT: Activated partial thromboplastin time
PT: Prothrombin time
HGB: Hemoglobin
HCT: Hematocrit
PLT: Platelet
NTD: Neural tube defect
